# Yolk–Shell Silicon–Carbon Anodes with Interconnected N-Doped Carbon Networks for Stable Lithium-Ion Storage

**DOI:** 10.3390/ma19112286

**Published:** 2026-05-28

**Authors:** Yi Zhou, Yi Zhang, Zhanhong Zhao, Yansen Qu, Jiajun Wu, Xueqin Ma, Xinghua Chang

**Affiliations:** 1 School of Minerals Processing and Bioengineering, Central South University, Changsha 410083, China; 235611071@csu.edu.cn (Y.Z.); yee_z10@csu.edu.cn (Y.Z.); 18839127902@163.com (Z.Z.); 245612083@csu.edu.cn (J.W.); 245612085@csu.edu.cn (X.M.); 2 Key Laboratory for Mineral Materials and Application of Hunan Province, School of Minerals Processing and Bioengineering, Central South University, Changsha 410083, China

**Keywords:** silicon-carbon anode, lithium-ion batteries, Si/C composites, N-doped carbon network, void-buffer structure

## Abstract

Silicon-based anodes are considered promising alternatives to graphite anodes owing to their high theoretical lithium-storage capacity and abundant reserves. However, silicon nanoparticle anodes are severely limited by large volume expansion, unstable interfacial chemistry, and poor electrical connectivity during repeated lithiation/delithiation. Herein, we develop a yolk–shell N-doped carbon network (NCN) strategy to construct Si@void@NCN composites. The optimized Si@void@NCN-1 achieves a balanced architecture between void buffering and carbon network integrity, delivering a high initial discharge capacity of 1245.5 mAh g^−1^ and an initial charge capacity of 735.8 mAh g^−1^. It also demonstrates stable long-term cycling performance, retaining a reversible capacity of 402.5 mAh g^−1^ after 500 cycles at 0.5 A g^−1^ with a capacity retention of 68.66%, and shows improved rate reversibility and electrode structural stability, with an electrode thickness increase of only 80.4% after rate cycling, much lower than that of densely carbon-coated Si@C. Kinetic analysis, post-cycling structural characterization, and in situ EIS further reveal that the yolk–shell void-buffering structure and the N-doped three-dimensional conductive network act synergistically to mitigate Si volume expansion, enhance structural stability, and facilitate electron/ion transport. This study emphasizes the importance of integrating buffering structures with Si/C composites, providing guidance for the rational design of advanced silicon-based electrode materials.

## 1. Introduction

With the rapid development of portable electronics, electric vehicles, and large-scale energy storage systems, the demand for lithium-ion batteries (LIBs) with higher energy density has continuously increased [1,2,3]. Owing to their high energy and power densities, suitable operating voltage, and low self-discharge, LIBs have become one of the most widely used electrochemical energy storage systems [4,5]. However, the limited theoretical specific capacity of commercial graphite anodes (372 mAh g^−1^) can hardly meet the requirements of next-generation high-energy-density batteries. In contrast, silicon has been regarded as a promising anode candidate because of its extremely high theoretical capacity, low lithiation potential, abundant reserves, and environmental friendliness. The development of high-performance silicon anodes can not only exploit the high-capacity advantage of silicon but also help reduce dependence on certain critical metal resources [6,7,8].

Nevertheless, silicon anodes suffer from severe volume expansion of up to ~300% during lithiation, followed by contraction during delithiation [9]. This repeated expansion and shrinkage can cause particle pulverization, conductive network failure, continuous rupture and reconstruction of the solid electrolyte interphase (SEI), and electrode delamination, ultimately leading to rapid capacity decay and reduced Coulombic efficiency [10,11]. In addition, the intrinsically poor electrical conductivity of silicon limits its electrochemical reaction kinetics, especially under high-rate conditions. Therefore, simultaneously mitigating volume variation, improving electron/ion transport, and stabilizing the electrode/electrolyte interface is critical for the practical application of silicon-based anodes [12,13].

Carbon materials have been widely used to modify silicon-based anodes because of their good electrical conductivity, chemical stability, and structural tunability. Silicon–carbon composites can improve the overall conductivity of silicon-based materials, buffer volume variation to some extent, and reduce the direct contact between silicon and electrolyte, thereby enhancing interfacial stability [14,15,16,17,18]. Typical silicon–carbon composite structures include core–shell, porous, embedded, and yolk–shell architectures. Core–shell structures improve electron transport and suppress side reactions by coating silicon particles with a carbon layer; however, their dense shells provide limited buffering space and are prone to fracture during long-term cycling due to repeated Si volume expansion [19,20,21]. Porous structures accommodate volume change through internal pores, but poor pore distribution or insufficient skeleton strength can lead to structural collapse and reduced conductive continuity [22]. Embedded structures enhance structural stability by dispersing silicon within a carbon matrix, yet their silicon content is often limited, restricting the full utilization of silicon’s high capacity [23,24]. Among these structural designs, yolk–shell architectures have attracted particular attention because they combine the advantages of carbon coating and internal void buffering. The void space between the Si core and carbon shell provides reserved room for Si volume expansion during lithiation while maintaining a relatively high Si loading, thereby overcoming the insufficient buffering space of dense core–shell structures. Meanwhile, the outer carbon shell helps preserve electronic contact, isolates the electrolyte, and promotes the formation of a relatively stable SEI layer, thereby reducing the risks of conductive network failure and continuous interfacial side reactions [25,26,27,28]. For example, raspberry-like yolk–shell silicon–carbon micro/nanospheres have been prepared through spray drying, pre-oxidation, carbonization, and HF etching [29]. Li et al. [30] used Sn as a sacrificial template, a uniform SnO_2_ layer was formed on the surface of Si nanoparticles by annealing, and the template was subsequently removed with concentrated HCl, avoiding the toxicity and corrosiveness associated with conventional HF etching. Meanwhile, a pitch-derived highly conductive carbon layer was used to construct a yolk–shell Si@void@C composite with tunable internal voids. In another work, Li et al. [8] uniformly dispersed ultrasmall amorphous silicon nanodots in carbon nanospheres and welded them onto the walls of a macroporous carbon framework using vertical graphene, constructing a hierarchically interconnected silicon–carbon anode with excellent half-cell performance.

Although significant progress has been made in the structural design and performance improvement of Si/C anodes, achieving an effective balance among volume buffering, conductive connection, interfacial stability, and synthetic simplicity remains a major challenge. This is mainly because the performance of yolk–shell Si/C composites strongly depends on the coordinated matching of void size, carbon shell thickness, and conductive network continuity [31]. Although void structures can accommodate silicon volume variation, insufficient void space cannot fully buffer expansion, whereas excessive void space may weaken structural integrity and electronic continuity. On the other hand, carbon layer thickness also requires precise regulation. A thin carbon layer may provide insufficient protection and conductive support, while an excessively thick carbon layer can reduce active silicon utilization and increase Li^+^ transport resistance [32,33]. Moreover, some high-performance yolk–shell structures still rely on complex templates, multistep etching, or delicate assembly processes, which are unfavorable for scalable production. Therefore, developing a facile strategy to construct silicon–carbon composites with tunable void size, appropriate carbon shell thickness, and continuous conductive networks remains an important direction for improving the overall performance of silicon-based anodes.

Based on these considerations, a yolk–shell silicon–carbon composite consisting of a Si core, internal void space, and an interconnected N-doped carbon network was constructed through a polystyrene (PS) sacrificial-template strategy, in situ aniline polymerization, and subsequent carbonization. The obtained material was denoted as Si@void@NCN. In this structure, PS decomposes and is removed during thermal treatment, generating an internal void between the silicon core and the outer carbon layer, which provides effective space for accommodating silicon expansion. Meanwhile, polyaniline (PANI) is converted into an interconnected N-doped carbon framework, which enhances electron transport and improves interfacial stability. By regulating the PS content and carbon precursor amount, the optimized Si@void@NCN-1 achieves a favorable balance among void size, carbon layer thickness, and conductive network continuity, thereby reducing structural degradation and improving Li^+^ transport kinetics during cycling. Benefiting from this architecture, Si@void@NCN-1 delivers a reversible capacity of 402.5 mAh g^−1^ after 500 cycles at 0.5 A g^−1^, with a capacity retention of 68.66%, demonstrating improved cycling stability and electrochemical reversibility. This work offers a feasible strategy for the rational design of durable Si/C anode materials.

## 2. Materials and Methods

### 2.1. Chemical Reagent

Silicon powder (Si, 99.999%, 20–200 nm) was purchased from Shanghai Xiaohuang Nano Technology Co., Ltd. (Shanghai, China); polystyrene (PS, (C_8_H_8_)_n_, Mw∼150,000), N,N-dimethylformamide (DMF, C_3_H_7_NO, analytical grade), Span 20 (C_18_H_34_O_6_, nonionic), aniline (C_6_H_5_NH_2_, analytical grade), ferrous sulfate heptahydrate (FeSO_4_⋅7H_2_O, analytical grade), and sodium alginate (C_6_H_9_NaO_7_, analytical grade) were purchased from Aladdin Biochemical Technology Co., Ltd. (Shanghai, China). Hydrogen peroxide (H_2_O_2_, 30%) and hydrochloric acid (HCl, analytical grade) were purchased from Sinopharm Chemical Reagent Co., Ltd. (Shanghai, China). Conductive carbon black (C, battery grade) was purchased from Timcal, Giornico, Switzerland. All chemicals were used as received without further purification.

### 2.2. Synthesis

#### 2.2.1. Synthesis of Si@PS Composite

Polystyrene (PS) was dissolved in N,N-dimethylformamide (DMF) to prepare a solution with a solid content of 15 wt%. The solution was kept in an oven at 80 °C overnight to ensure complete dissolution. Afterward, the solution was stirred continuously while Si powder was added. The mass ratios of PS to Si were controlled at 3, 4 and 5. The mixture was stirred overnight to achieve uniform dispersion of Si. Subsequently, the mixture was injected into deionized water via pneumatic atomization. During this process, rapid precipitation and solidification of PS occurred through solvent–nonsolvent exchange, leading to the formation of Si@PS composite particles. The product was vacuum-dried, crushed, and sieved to obtain the Si@PS composite. After sieving through a 300-mesh screen, the resulting Si@PS intermediate particles had a size range of 1–5 μm.

#### 2.2.2. Synthesis of Si@PS@PANI Composite

For the preparation of Si@PS@PANI, 100 mL of 1 mol L^−1^ HCl solution was used as the reaction medium. Aniline monomer was added in amounts of 0.5, 1.0, and 1.5 g, respectively. After uniform stirring, 0.1 g of Span 20 was added as a surfactant, followed by the addition of 1 g of Si@PS composite. The mixture was dispersed using a high-speed disperser for 30 min to ensure homogeneity. Then, 1 mL of 0.1 mol L^−1^ FeSO_4_ solution (prepared in 0.05 mol L^−1^ HCl) was added. At 5 °C, 50 mL of 5 wt% H_2_O_2_ solution was slowly dropped into the mixture. Using H_2_O_2_ as the oxidant and Fe^2+^ as the catalyst, the in situ oxidative polymerization of aniline was initiated, forming a PANI coating on the Si@PS surface. After the dropwise addition, the reaction mixture was stirred in a 5 °C cold-water bath for 1 h and then transferred to room temperature for another 12 h. The product was collected by centrifugation and dried in a vacuum oven at 60 °C to obtain the Si@PS@PANI composite.

#### 2.2.3. Synthesis of Si@void@NCN Composite

The Si@PS@PANI composite was subjected to programmed calcination. Specifically, the sample was first heated to 250 °C at 2 °C min^−1^ and held for 90 min, then to 390 °C at 1 °C min^−1^ and held for 60 min under strong gas purging, and finally to 800 °C at 5 °C min^−1^ and held for 120 min. After the thermal treatment, the final product (Si@void@NCN) was obtained. Depending on the amount of aniline added, three samples with different carbon contents were prepared, which are referred to as Si@void@NCN-0.5, Si@void@NCN-1, and Si@void@NCN-1.5, respectively.

### 2.3. Material Characterization

The morphology and elemental distribution of the samples were characterized by scanning electron microscopy (SEM, ZEISS Sigma 300, Oberkochen, Germany) equipped with an Xplore 30 energy-dispersive X-ray spectroscopy (EDS) detector at an accelerating voltage of 30 kV. Transmission electron microscopy (TEM) and high-resolution transmission electron microscopy (HRTEM) were performed on a JEOL JEM-F200 (Akishima, Japan) operated at 200 kV, equipped with a JED-2300T EDS system. Powder X-ray diffraction (XRD) patterns were collected using a TD-3500 X-ray diffractometer (Dandong Tongda Technology Co., Ltd., Dandong, China) with Cu Kα radiation at 35 kV. X-ray photoelectron spectroscopy (XPS) was carried out on a Thermo Scientific K-Alpha spectrometer (Waltham, MA, USA) with a monochromatic Al Kα X-ray source. Thermogravimetric analysis (TGA) was performed using a STA 449 F3 Jupiter instrument (NETZSCH, Selb, Germany) from room temperature to 900 °C under an air atmosphere at a heating rate of 10 °C min^−1^. Raman spectra were recorded on a Renishaw InVia Qontor Raman spectrometer (Renishaw, Wotton-under-Edge, UK) with a 532 nm laser to analyze the carbon structure of the samples. Fourier transform infrared spectroscopy (FTIR) spectra were obtained using a Nicolet iS50 FTIR spectrometer (Thermo Scientific, Waltham, MA, USA) to identify the functional groups of the samples.

### 2.4. Electrochemical Measurements

The electrochemical performance of the samples as anodes for LIBs was evaluated using CR2032 coin-type half-cells (Canrd, Dongguan, China). The active material, Super P, and sodium alginate (SA) were mixed at a weight ratio of 7:2:1 in deionized water by grinding and stirring. The obtained slurry was then cast onto Cu foil and dried under vacuum at 60 °C. The electrodes were punched into disks with a diameter of 12 mm, and the mass loading of the active material was 0.5–1.0 mg cm^−2^. Li metal was used as the counter/reference electrode, Celgard 2500 polypropylene membrane was used as the separator, and 1 M LiPF_6_ in EC/DEC (1:1, *v*/*v*) containing 20 wt% FEC and 1 wt% VC was used as the electrolyte. The half-cells were assembled in an Ar-filled glovebox.

Cyclic voltammetry (CV) was performed on a Gamry electrochemical workstation at a scan rate of 0.1 mV s^−1^. Electrochemical impedance spectroscopy (EIS) was conducted on a CHI 660E electrochemical workstation at open-circuit voltage over a frequency range of 100 kHz to 0.01 Hz. Galvanostatic charge–discharge (GCD) and galvanostatic intermittent titration technique (GITT) measurements were carried out using a LAND battery testing system. All electrochemical measurements were performed at room temperature. The distribution of relaxation times (DRT) analysis was performed using EIS Analysis Tool 3.0 developed by Xin Liu.

## 3. Results and Discussion

### 3.1. Materials Synthesis and Microstructure

**Figure 1:**
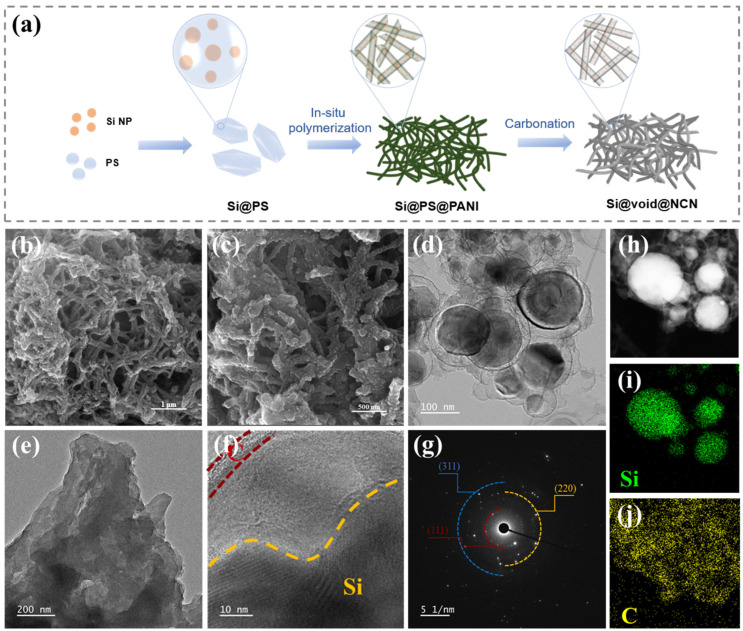
(**a**) Schematic illustration of the synthesis processes and structural diagram of Si@void@NCN; (**b**,**c**) SEM images of Si@void@NCN-1; (**d**–**j**) TEM, HRTEM images and STEM-EDS mappings of Si@void@NCN-1.

Si@PS precursor particles were first prepared by dispersing Si powder in a PS/DMF solution, followed by pneumatic atomization into deionized water, where solvent–nonsolvent exchange induced rapid PS precipitation around Si. Subsequently, PANI was coated onto Si@PS via in situ oxidative polymerization of aniline, and the resulting Si@PS@PANI composite was carbonized under an Ar atmosphere to remove PS and convert PANI into an N-doped carbon network, yielding the final Si@void@NCN composite (Figure 1a).

Final carbonized samples were characterized by electron microscopy to examine their microstructure and elemental distribution (Figure 1b,c). The directly carbon-coated Si@C sample exhibits a dense core–shell structure, where the carbon layer adheres closely to the Si surface and particles are compactly packed without obvious voids or interconnected channels (Figure S1a). In contrast, after PS introduction, the Si@void@NCN samples display a porous network morphology with interconnected carbon frameworks and abundant pores/channels. Although the Si@PS precursor appears as large aggregated particles (Figure S1b), the final product evolves into a loose network after PANI coating and thermal treatment. Thus, PS not only serves as a removable template for void formation but also regulates the spatial arrangement of the PANI-derived carbon layer, promoting a three-dimensional conductive network.

TEM images reveal that with an appropriate amount of PS, a clear gap forms between the Si core and carbon shell, creating an internal void (Figure S2c). Increasing PS content enlarges the void size, indicating that PS effectively tunes the buffering space (Figure 1d). The outer carbon layer is not a dense coating but consists of interconnected sheets/frameworks with good porosity and connectivity. This structure accommodates Si volume expansion during lithiation while facilitating electrolyte infiltration and Li^+^ transport. HRTEM shows clear lattice fringes in the Si region and an amorphous feature in the carbon region (Figure 1f). The selected area electron diffraction (SAED) pattern further exhibits the (111), (220), and (311) planes of crystalline Si, respectively (Figure 1g). EDS mapping shows Si mainly in the inner particle region, while C is uniformly distributed over the outer layer and interparticle connections, confirming a continuous carbon framework.

### 3.2. Characterization of Si@void@NCN Composite Anodes

**Figure 2:**
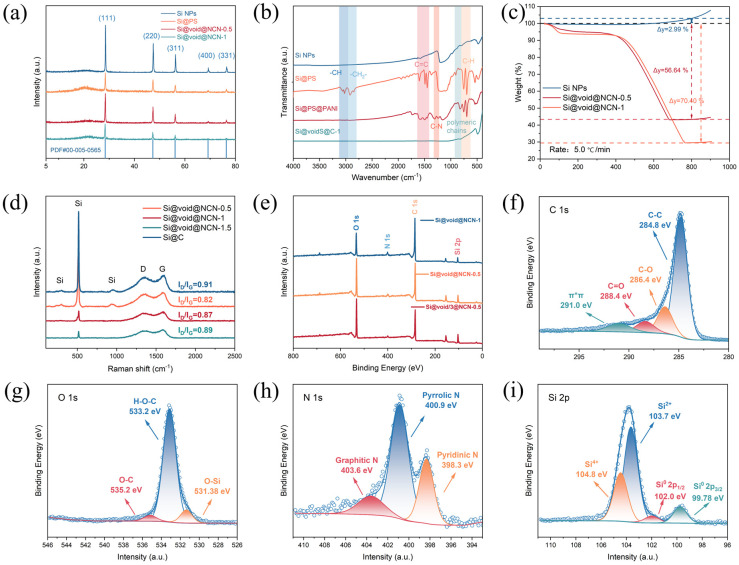
Structural and chemical characterization: (**a**) XRD patterns; (**b**) FTIR spectra; (**c**) TGA curves; (**d**) Raman spectra; (**e**) XPS survey spectrum of Si@void@NCN-1; High-resolution XPS spectra of (**f**) C 1s, (**g**) O 1s, (**h**) N 1s, and (**i**) Si 2p of Si@void@NCN-1.

To identify the crystalline phase and evaluate whether the synthesis process affected the Si crystal structure, XRD measurements were performed, and the corresponding patterns are shown in Figure 2a. All samples exhibit characteristic diffraction peaks at 28.4°, 47.3°, 56.1°, 69.1°, and 76.4°, which can be indexed to the (111), (220), (311), (400), and (331) planes of crystalline Si (PDF#00-005-0565), respectively. In the Si@PS series, a broad low-angle peak appears with increasing PS content, confirming amorphous PS coating without altering the Si phase (Figure S3). The Si@void@NCN-1 samples retain all Si peaks along with a low-angle broad background, indicating an amorphous carbon coating after high-temperature treatment.

The successful construction of the Si@PS intermediate was further verified by FTIR spectroscopy (Figure 2b). Compared with pristine Si, Si@PS displays new absorption bands at approximately 3023 cm^−1^, assigned to the aromatic C–H stretching vibration, and at 2917 and 2850 cm^−1^, corresponding to the –CH_2_– stretching vibrations. In addition, the characteristic aromatic C=C vibrations at around 1600 and 1492 cm^−1^ are clearly observed. The emergence of these PS-related signals confirms that PS was successfully introduced onto the Si particle surface, providing the sacrificial polymer layer for subsequent void formation [34,35]. After subsequent in situ polymerization of aniline, characteristic absorption bands of PANI are observed in the Si@PS@PANI sample. The peak at 819 cm^−1^ suggests the presence of di- and/or trisubstituted aromatic rings and the formation of polymeric chains [36]. The absorption bands at approximately 1583 and 1496 cm^−1^ are assigned to the vibrations of quinoid and benzenoid structures in PANI, while the peak at around 1350 cm^−1^ corresponds to the C-N stretching vibration [37]. After high-temperature calcination, PS/PANI-related signals in Si@void@NCN-1 are largely eliminated, leaving only weak Si-O bands, indicating PS decomposition and PANI carbonization. This transformation is essential for generating the internal void space and converting the PANI coating into a conductive carbon framework.

The carbon content in the Si@void@NCN composites was quantified by TGA (Figure 2c). Silicon nanoparticles (Si NPs) show only a slight weight gain during heating, which is mainly attributed to surface oxidation. In contrast, the Si@void@NCN samples exhibit a pronounced weight loss in the range of 400–750 °C, corresponding to the oxidative decomposition of the carbon component. Increasing the amount of aniline from 0.5 to 1 raises the carbon content from 56.64% to 70.40% (Figure 2c), confirming that aniline dosage effectively regulates PANI-derived carbon content. Raman spectroscopy was used to evaluate carbon structural ordering. All composites show typical D and G bands. The ID/IG values for Si@C, Si@void@NCN-0.5, Si@void@NCN-1, and Si@void@NCN-1.5 are 0.91, 0.82, 0.87, and 0.89, respectively (Figure 2d). The lower ID/IG values of void-containing composites compared with Si@C indicate higher structural ordering of the PANI-derived carbon network, which is beneficial for constructing continuous electron transport pathways [38].

The surface composition and chemical states of Si@void@NCN-1 were further investigated by XPS (Figure 2e–i). The survey spectrum confirms the presence of C, O, N, and Si, indicating that PANI was successfully converted into an N-doped carbon layer on the composite surface after carbonization. In the high-resolution C 1s spectrum (Figure 2f), the peaks corresponding to C–C/C=C, C–O, and C=O confirm the formation of a carbon framework containing oxygen-containing functional groups [39]. The N 1s spectrum is deconvoluted into pyridinic N, pyrrolic N, and graphitic N [10]. Pyridinic and pyrrolic N are generally associated with interfacial active sites for Li^+^ adsorption and charge transfer, whereas graphitic N suggests the incorporation of N atoms into the carbon framework, which may contribute to electronic transport. The Si 2p spectrum shows elemental Si alongside a small amount of SiO_x_, indicating a thin surface oxide layer while the internal Si core remains intact [40,41]. Collectively, these results demonstrate the successful construction of Si@void@NCN composites with a crystalline Si core, internal void space, and an N-doped conductive carbon framework. This confirms the effectiveness of the PS sacrificial template and PANI-derived carbon strategy in regulating the spatial structure and carbon network configuration of the composite.

### 3.3. Electrochemical Performances of Si@void@NCN Composite Anodes

With the purpose of exploring the Li-storage behavior of Si NPs and PANI, GCD measurements were performed at 0.1 A g^−1^. As shown in Figure S4, Si NPs delivered a high initial charge specific capacity of 2704.4 mAh g^−1^, but suffered from rapid capacity fading during subsequent cycling, indicating poor structural stability caused by severe volume variation. In contrast, PANI exhibited much better cycling stability but a low initial charge capacity of only 240.2 mAh g^−1^, limiting its contribution to overall energy storage. Therefore, constructing Si/C composite anodes is essential to combine the high capacity of Si with the structural stability and conductive buffering effect of carbonaceous materials. The theoretical specific capacity of the Si/C composite was calculated using Equation (1):

(1) $$C_{\mathrm{Si}/C}\;=\;\omega_{\mathrm{Si}}C_{\mathrm{Si}}\;+\;\omega_{C}C_{C}$$

where $C_{Si/C}$ is the theoretical specific capacity of the Si/C composite; $\omega_{\mathrm{Si}}$ and $\omega_{C}$ are the mass fractions of Si and carbon, respectively; $C_{Si}$ is the specific capacity of Si; and $C_{C}$ is the specific capacity of the carbon material.

Based on the TG results, the Si and carbon contents in Si@void@NCN-0.5 are 43.36% and 56.64%, respectively, while those in Si@void@NCN-1 are 29.60% and 70.40%, respectively. Using Equation (1), the theoretical specific capacities of Si@void@NCN-0.5 and Si@void@NCN-1 were calculated to be 1308.68 and 969.60 mAh g^−1^, respectively. With the purpose of exploring lithium storage behavior of Si@void@NCN, GCD measurements were performed at 0.1 A g^−1^. With increasing PS content, the initial discharge capacity decreases due to reduced active Si contribution (Figure S5a,b). Si@void/4@NCN achieves the optimal balance, delivering an initial capacity of 1103.1 mAh g^−1^ with 68.54% retention after 100 cycles and 756.1 mAh g^−1^ remaining (Figure S5c).

**Figure 3:**
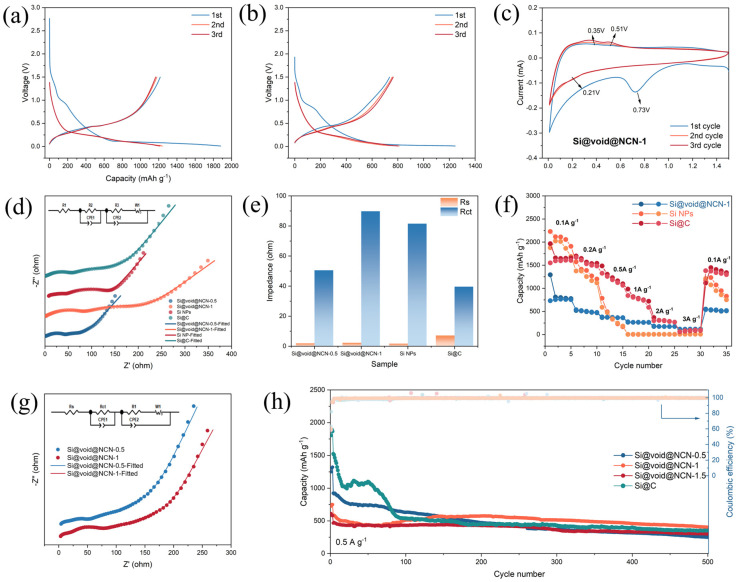
Lithium-ion storage performances: Typical charge/discharge curves of (**a**) Si@void@NCN-0.5 and (**b**) Si@void@NCN-1; (**c**) CV curves; (**d**) Nyquist plots; (**e**) Contribution ratios of Rs and Rct; (**f**) Rate performance; (**g**) Nyquist plots after rate test; (**h**) Cycling performance at 0.5 A g^−1^ (the first three cycles were tested at 0.1 A g^−1^).

Accordingly, the optimized PS-Si mass ratio is determined to be 4. Figure 3a,b show the first three charge–discharge profiles of Si@void@NCN samples with different carbon contents at a fixed PS:Si mass ratio of 4. All samples exhibit typical Si-based anode behavior, while the low-voltage plateau represents Si-Li alloying [42]. GCD results show that the initial charge specific capacities of Si@void@NCN-0.5 and Si@void@NCN-1 are 1216.3 and 735.8 mAh g^−1^, respectively. The initial Coulombic efficiency (ICE) of Si@void@NCN-1 is 59.08%, which is mainly attributed to irreversible lithium consumption during the first lithiation, including SEI formation, irreversible lithiation of surface SiO_x_, and lithium trapping in the defect-rich N-doped carbon layer. The Coulombic efficiency subsequently recovers in the following cycles. Strategies such as pre-lithiation, electrolyte optimization, or fine-tuning of the carbon network structure may be explored in future work to improve ICE while maintaining the excellent cycling stability. The charge–discharge profiles of other Si@void@NCN samples with different carbon contents are provided in Figure S6. As depicted in Figure 3c, the initial three CV plots of the Si@void@NCN-1 electrode were recorded with a scan rate of 0.1 mV s^−1^. In the first cathodic scan, reduction peaks at 0.73 V (SEI formation) [29] and 0.21 V (Si-Li alloying to amorphous Li_x_Si) are observed. After the first cycle, the 0.73 V peak nearly disappears, indicating stable SEI establishment and suppressed side reactions. In the anodic scan, two oxidation peaks at 0.35 and 0.51 V correspond to stepwise delithiation of Li_x_Si [43]. The overlapping second and third cycles indicate good reversibility and interfacial stability [10,30].

EIS measurements and equivalent circuit fitting were performed to evaluate the interfacial charge-transfer behavior of the electrodes. As shown in Figure 3d,e, Nyquist plots consist of two semicircles in the high-to-medium frequency region and an inclined line in the low-frequency region, corresponding to interfacial processes and Li^+^ diffusion, respectively [44,45]. All samples have similar Rs but significantly different Rct. Si@C and Si@void@NCN-0.5 exhibit lower Rct due to favorable interfacial contact, while Si@void@NCN-1 shows higher initial Rct from its thicker carbon coating, which nonetheless provides better cycling stability. The rate capability was evaluated at different current densities, as shown in Figure 3f. Si@void@NCN-1 delivers capacities of 731.5, 537.2, 381.3, 276.0, 183.0, and 118.2 mAh g^−1^ at 0.1–3 A g^−1^, recovering to 538.9 mAh g^−1^ at 0.1 A g^−1^. Si NPs and Si@C show rapid decay or capacity loss at high rates, confirming the advantage of the yolk–shell N-doped network. After the rate test, EIS measurements further show that Si@void@NCN-0.5 impedance increases markedly after cycling, whereas Si@void@NCN-1 exhibits lower impedance, indicating better electrode integrity and interfacial stability, consistent with its superior rate performance.

Figure 3h shows the cycling characteristics of the composites at 0.5 A g^−1^. Si NPs capacity decays sharply within 100 cycles, while carbon coating and void structure significantly improve cycling stability. Among the three Si@void@NCN samples, Si@void@NCN-1 shows the best performance: 402.5 mAh g^−1^ after 500 cycles (68.66% retention from the 4th cycle). Si@void@NCN-0.5 has higher initial capacity but faster fading due to insufficient buffering from its thinner carbon layer. Si@void@NCN-1.5 retains only 300 mAh g^−1^ (64.28% retention). Its excessive carbon layer reduces void buffering and stress release efficiency, leading to gradual structural degradation. Although the high carbon content contributes to structural stability, the inferior performance of the sample prepared with another carbon source indicates that the superior electrochemical behavior of Si@void@NCN-1 mainly arises from the synergistic effect of the optimized void-buffering structure and PANI-derived N-doped carbon network, rather than carbon content alone. (Figure S7a) To evaluate the effect of carbonization temperature, a control sample carbonized at 700 °C was investigated. It delivered initial discharge/charge capacities of 1458.2 and 840.9 mAh g^−1^, and its charge capacity dropped to 243.9 mAh g^−1^ after 500 cycles—far inferior to that of the 800 °C sample. This indicates that insufficient carbonization at a lower temperature results in a less conductive network. Therefore, under the present conditions, 800 °C offers a better balance between conductivity and structural preservation (Figure S7b).

TEM was employed to directly examine the microstructure of the electrode material after rate testing, while SEM was used to measure the cross-sectional thickness of the electrodes after rate cycling. As shown in Figure S8, Si@void@NCN-1 maintains a relatively intact structure after rate cycling, with no obvious particle fracture or densification. Cross-sectional views show a thickness increase from 10 μm to 18.04 μm (80.4%), with uniform morphology, good adhesion, and no delamination or cracks, indicating good mechanical stability. In contrast, the Si@C electrode thickens to 30.35 μm (203.5% increase), with a looser, uneven morphology and local swelling. Thus, Si@void@NCN-1 exhibits much lower swelling and better structural retention. These results demonstrate that the void structure and conductive carbon framework can effectively buffer Si volume variation, preserve electrode integrity, and mitigate particle pulverization and interfacial degradation during high-rate cycling. This structural stability is consistent with the improved rate capability and cycling performance of Si@void@NCN-1 [46,47].

### 3.4. Electrochemical Behavior of Si@void@NCN Composites Anodes

**Figure 4:**
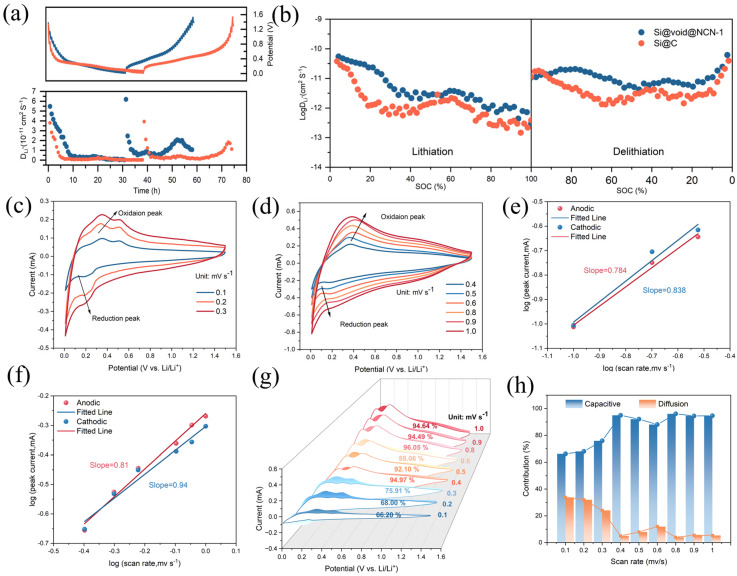
(**a**) GITT curve and corresponding $D_{{\mathrm{Li}}^{+}}$; (**b**) $D_{{\mathrm{Li}}^{+}}$ of Si@void@NCN-1 and Si@C during lithiation/delithiation; (**c**,**d**) CV curves at different scan rates; (**e**,**f**) Relationship between log(i) and log(v); (**g**) CV profiles of the pseudocapacitance ratio of Si@void@NCN-1; (**h**) Quantitative ratios of capacitive and diffusion-controlled contributions.

To investigate the evolution of Li-ion diffusion kinetics, the galvanostatic intermittent titration technique (GITT) was employed to evaluate the Li-ion diffusion coefficient [4,48], and the corresponding profiles are shown in Figure 4a,b. According to Fick’s second law, the Li-ion diffusion coefficient ($D_{{\mathrm{Li}}^{+}}$) can be calculated using Equation (2).

(2) $$D_{{\mathrm{Li}}^{+}}=\frac{4}{\pi \tau }{\left(\frac{m_{B}V_{M}}{M_{B}S}\right)}^{2}{\left(\frac{∆E_{S}}{∆E_{t}}\right)}^{2}$$

where τ is the pulse duration set by the testing program; $m_{B}$ is the mass of the Si/C active material; $V_{M}$ is the molar volume of the Si/C material; $M_{B}$ is the molar mass of the Si/C material; S is the contact area between the electrode and electrolyte, which is approximated by the geometric area of the electrode; and $∆E_{S}$ and $∆E_{t}$ are the voltage changes during the relaxation period and the current pulse, respectively.

Because pronounced side reactions occur during the initial charge–discharge process, $D_{{\mathrm{Li}}^{+}}$ was calculated from the second cycle. As shown in Figure 4a,b, the variation in the lithium-ion diffusion coefficient ($D_{{\mathrm{Li}}^{+}}$) at different states of charge (SOC) indicates that Si@void@NCN-1 exhibits higher $D_{{\mathrm{Li}}^{+}}$ values than Si@C over most of the lithiation and delithiation ranges, suggesting improved ${Li}^{+}$ transport kinetics. In the deep lithiation region, where Si undergoes severe volume expansion, Si@void@NCN-1 maintains a $D_{{\mathrm{Li}}^{+}}$ of 1.22 × 10^−12^ cm^2^ s^−1^ at around 80.87% SOC, much higher than that of Si@C at a similar SOC (2.63 × 10^−13^ cm^2^ s^−1^). During the main delithiation region, Si@void@NCN-1 also shows a higher $D_{{\mathrm{Li}}^{+}}$ of 5.74 × 10^−12^ cm^2^ s^−1^, compared with 1.98 × 10^−12^ cm^2^ s^−1^ for Si@C. This enhancement can be attributed to the internal void structure and conductive carbon network, which improve electrolyte infiltration, provide accessible ${Li}^{+}$ transport pathways, and reduce mass-transfer resistance, thereby supporting the superior rate performance of Si@void@NCN-1.

To further investigate the Li-storage kinetics of the electrode material, cyclic voltammetry (CV) measurements were conducted at different scan rates. The reaction kinetics were analyzed based on the relationship between the peak current (i) and the scan rate (v):

(3) $$i\;=\;a\nu^{b}$$

Taking the logarithm of both sides gives:

(4) log(i) = b log(ν) + log(a)

where the b-value is used to characterize the kinetic behavior of the electrochemical reaction. b-value close to 0.5 indicates a diffusion-controlled process, whereas b-values close to 1 suggests a surface capacitive-controlled process. The particular proportions from the diffusion-controlled and pseudocapacitance mechanisms are quantitatively calculated by the formula below:

(5) $$i(v)\;=\;k_{1}v^{1/2}\;+\;k_{2}v$$

(6) $$i(v)/v^{1/2}=k_{1}+k_{2}v^{1/2}$$

As shown in Figure 4c, Si@void@NCN-1 exhibits a cathodic peak at ~0.35 V (Si-Li alloying to Li_x_Si) and two anodic peaks at ~0.30 and 0.45 V, indicating good reversibility. As scan rate increases (Figure 4d), redox peaks broaden and overlap; at 0.4–1.0 mV s^−1^, peaks merge due to shortened reaction time and increased polarization. Thus, kinetic analysis was divided into low (0.1–0.3 mV s^−1^) and high (0.4–1.0 mV s^−1^) scan rate regions. Figure 4e,f shows that in the low scan rate region, b-values of anodic and cathodic peaks are 0.784 and 0.838, indicating mixed diffusion-capacitive control. In the high scan rate region, b-values increase to 0.81 and 0.94, suggesting dominance of surface-controlled kinetics, especially during lithiation. Capacitive contribution (Figure 4g) increases from 66.20% to 94.64% as scan rate increases from 0.1 to 1.0 mV s^−1^, the detailed pseudocapacitive contribution at different scan rates is shown in Figure S9. This result suggests that Si@void@NCN-1 possesses a pronounced fast surface-controlled storage behavior, especially under high-rate conditions. Together with EIS and GITT results, Si@void@NCN-1 facilitates electrolyte infiltration, Li^+^ transport, and electron conduction, improving electrode kinetics.

### 3.5. Interfacial Evolution of Si@void@NCN Composite Anodes

**Figure 5:**
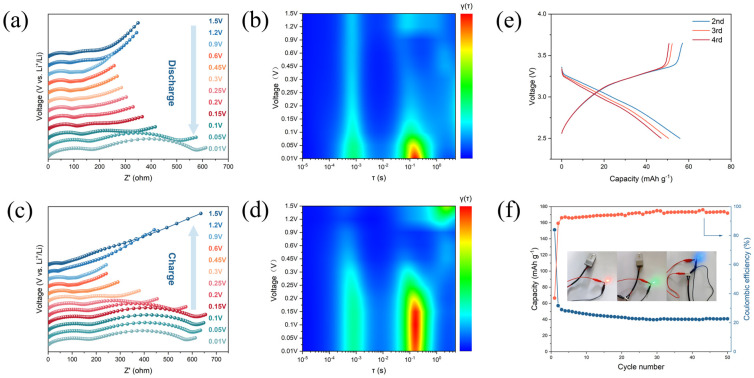
(**a**) In situ EIS spectra of Si@void@NCN-1 during the discharge process, each colored curve corresponds to the potential labeled with the same color on the right; (**b**) DRT analysis at different potentials during discharge process; (**c**) In situ EIS spectra of Si@void@NCN-1 during the charge process; (**d**) DRT analysis at different potentials during charge process; (**e**) Charge–discharge profiles of the Si@void@NCN-1//LiFePO_4_ full cell; (**f**) Cycling performance and Coulombic efficiency of the Si@void@NCN-1//LiFePO_4_ full cell, with inset photographs showing LEDs powered by the assembled full cell.

To investigate electrode interfacial evolution during lithiation/delithiation, in situ EIS was performed at different potentials (Figure 5a,c). During discharge, the in situ EIS spectra show a gradual increase in impedance as the potential decreases. The impedance changes only slightly in the range of 0.6–1.5 V, which mainly corresponds to electrode wetting, SEI formation, and surface reactions. Below 0.2 V, especially at 0.05–0.01 V, the impedance increases markedly, indicating enhanced charge-transfer and ion-diffusion resistance caused by deep Si lithiation, Li_x_Si formation, and increased interfacial stress. During charge, the impedance gradually decreases with increasing potential, which is attributed to the stepwise delithiation of Li_x_Si, Si volume contraction, and partial stress release. The relatively small impedance variation in the range of 0.3–0.6 V indicates a stable delithiation process and good structural continuity of the Si@void@NCN-1 electrode [49,50]. These results demonstrate that the void-containing carbon coating alleviates volume variation, stabilizes the interface, and improves reaction kinetics.

DRT analysis was further applied to deconvolute the overlapping relaxation processes in the in situ EIS spectra. The time-constant regions are generally associated with interfacial responses, charge transfer, and slower diffusion-related processes, respectively [51,52,53].The relationship between DRT and EIS data in the medium-to-high-frequency region is expressed as follows:

(7) $$Z_{\exp}\left(f\right)\;=\;\int_{-\infty }^{\infty }\frac{\gamma (\ln\tau )}{1\;+\;i2\pi f\tau }\mathrm{dln}\tau$$

where $Z_{\exp}\left(f\right)$ is the impedance measured at frequency f, τ is the time constant, and γ(lnτ) is the DRT function with respect to lnτ. To distinguish the contributions of different kinetic processes in the in situ EIS spectra, DRT analysis was performed on the EIS data collected at different potentials. Figure 5b shows the discharge process, and Figure 5d shows the charge process. Additional DRT curves showing the peak evolution during discharge and charge processes are provided in Figure S10. Generally, the short-time-constant region (10^−4^–10^−3^ s) corresponds to ohmic response, interfacial film processes, and fast surface reactions; the intermediate region relates to charge transfer; and the long region (10^−2^–10^−1^ s) is associated with solid-state diffusion, phase evolution, and mass-transfer limitations during deep lithiation/delithiation. During discharge, a weak-to-moderate peak appears in the short-time-constant region throughout the process with only slight shifts, indicating a stable fast interfacial process (e.g., surface film, electrode/electrolyte contact, fast surface Li storage) [51]. In contrast, the peak in the intermediate-to-long region intensifies as the discharge potential decreases, especially at 0.1–0.01 V, reaching a maximum near 0.01 V. This indicates increasingly limited charge transfer and solid-state diffusion during deep lithiation due to Li_x_Si formation, which increases mass-transfer and interfacial reaction resistance. During charge, the DRT spectra also show short and intermediate-to-long response regions, but their evolution differs. At the initial low-potential stage (0.01–0.15 V), the intermediate response remains strong, indicating that early delithiation is still affected by interfacial reaction and local diffusion from the highly lithiated Li_x_Si. As the potential increases to 0.2–0.6 V, this response gradually weakens, suggesting partial stress release and reduced interfacial/transport resistance. At 1.2–1.5 V, the long-time-constant response increases again and shifts to longer times, likely due to enhanced polarization, intensified ion-concentration gradients, and sluggish transport. Combined with the pseudocapacitive analysis, these results indicate that Li storage in Si@void@NCN-1 involves both fast surface reactions and bulk diffusion, contributing to its improved rate capability and cycling reversibility.

As a proof-of-concept demonstration, a Si@void@NCN-1//LiFePO_4_ full cell was assembled to evaluate the practical feasibility of the anode. As shown in Figure 5e, the full cell exhibits typical charge–discharge profiles during the 2nd–4th cycles, with the discharge capacity decreasing from approximately 57.1 to 50.7 mAh g^−1^ during the initial stabilization process. The cycling performance in Figure 5f shows that the capacity decays rapidly in the first few cycles and then gradually stabilizes, retaining approximately 40.8 mAh g^−1^ after 50 cycles. The initial capacity loss is mainly associated with continued SEI formation on the Si-based anode, possible nonuniform prelithiation, irreversible Li consumption, and imperfect capacity matching between the cathode and anode. In addition, the assembled full cell was able to power LEDs at different voltages, confirming its practical output capability.

## 4. Conclusions

In summary, we utilized a void-containing silicon–carbon anode, Si@void@NCN, which features an internal buffer space and an interconnected N-doped carbon network and was fabricated via a PS sacrificial-template strategy, in situ polyaniline coating, and subsequent carbonization. The optimized Si@void@NCN-1 achieves a well-balanced structure that effectively accommodates Si volume variation, maintains electrode integrity, and facilitates Li^+^/electron transport during repeated charge–discharge processes. As a result, the Si@void@NCN-1 electrode delivers a reversible capacity of 402.5 mAh g^−1^ after 500 cycles at 0.5 A g^−1^, corresponding to a capacity retention of 68.66%. Moreover, its electrode thickness increase after rate cycling is only 80.4%, which is much lower than that of the densely carbon-coated Si@C electrode (203.5%), confirming the improved structural stability and durability of the designed Si/C anode. This work demonstrates that coupling void-buffer engineering with an interconnected N-doped conductive carbon network is an effective strategy for developing stable and durable Si/C anodes for lithium-ion batteries.

## Data Availability

The original contributions presented in this study are included in the article/Supplementary Materials. Further inquiries can be directed to the corresponding author.

## Supplementary Materials

The following supporting information can be downloaded at: https://www.mdpi.com/article/10.3390/ma19112286/s1, The Supporting Information is freely available. In addition, it contains several graphs that support analysis. Figure S1: SEM images of (a) Si@C, (b) Si@PS, (c) Si@void@NCN-0.5, (d) Si@void@NCN-1.5; Figure S2: TEM images of (a) Si@C, (b) Si@PS, (c) Si@void@NCN-0.5; Figure S3: XRD patterns of samples with different PS ratios; Figure S4: (a,b) Charge–discharge profiles and cycling performance of Si NPs and PANI at a current density of 100 mA g^−1^: (a,b) Si NPs; (c,d) PANI; Figure S5: Samples with different PS coating ratios: (a) charge–discharge profiles, (b) capacity retention, and (c) cycling performance; Figure S6: Charge–discharge profiles and cycling performance of (a) Si@void@NCN-1.5, (b) Si NPs; Figure S7: (a) Cycling performance comparison of the PAN-derived control sample with the same Si@PS ratio and carbon-source feeding amount, (b) Cycling performance of Si@void@NCN-1 carbonized at 700 °C; Figure S8: Post-rate-test morphology: (a) TEM image of the material, (b) cross-sectional image of the Si@void@NCN-1 electrode, and (c) cross-sectional image of the Si@C electrode; Figure S9: (a–i) The detailed pseudocapacitive contribution at different scan rates; Figure S10: (a) The DRT curves during discharge of Si@void@NCN-1, and (b) the DRT curves during charge of Si@void@NCN-1.
