# Supplementary material for: Yolk–Shell Silicon–Carbon Anodes with Interconnected N-Doped Carbon Networks for Stable Lithium-Ion Storage
Source: Materials (Basel). 2026 May 28;19(11):2286. doi: 10.3390/ma19112286 (PMC13257925; doi:10.3390/ma19112286)
Supplement: Supplementary file 1 [file materials-19-02286-s001.zip › materials-4332985-supplementary.pdf]

# Supplementary Material

# Yolk–Shell Silicon–Carbon Anodes with Interconnected N-Doped Carbon Networks for Stable Lithium-Ion Storage

**Yi Zhou [1], Yi Zhang [1], Zhanhong Zhao [1], Yansen Qu [1], Jiajun Wu [1], Xueqin Ma [1] and Xinghua Chang [1,2,*]**

[1] School of Minerals Processing and Bioengineering, Central South University, Changsha 410083, China; 235611071@csu.edu.cn (Y.Z.); yee_z10@csu.edu.cn (Y.Z.); 18839127902@163.com (Z.Z.); 245612083@csu.edu.cn (J.W.); 245612085@csu.edu.cn (X.M.)

[2] Key Laboratory for Mineral Materials and Application of Hunan Province, School of Minerals Processing and Bioengineering, Central South University, Changsha 410083, China

* Correspondence: changxinghua@csu.edu.cn

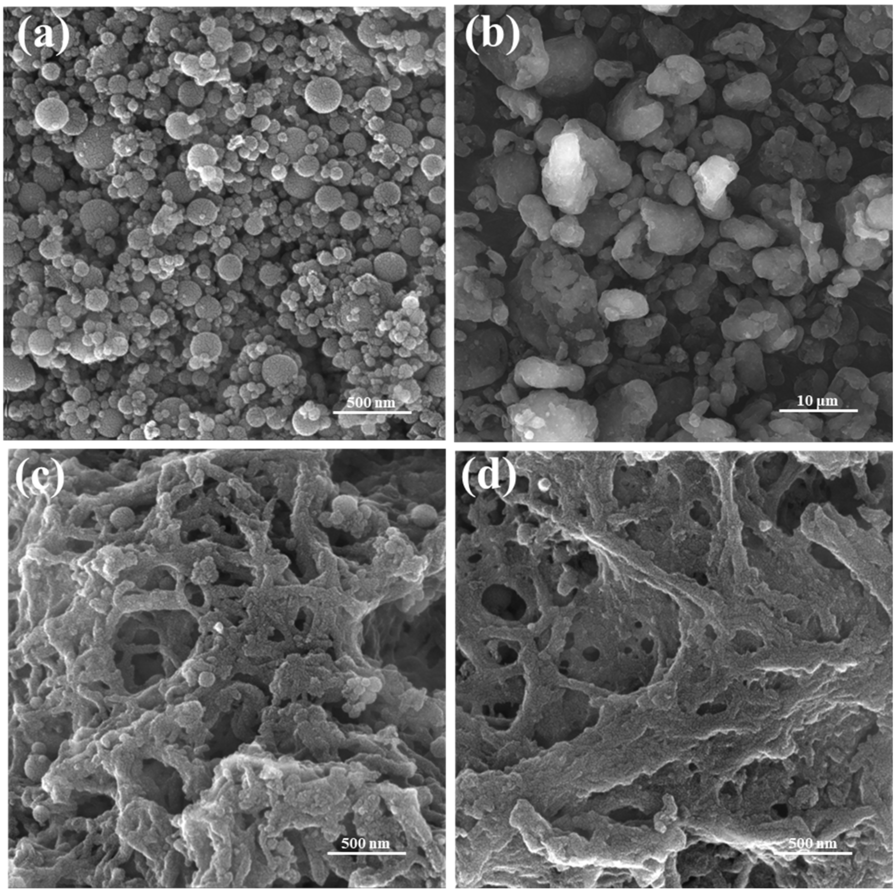


**Figure S1.** SEM images of (a) Si@C, (b) Si@PS, (c) Si@void@NCN-0.5, (d) Si@void@NCN-1.5.

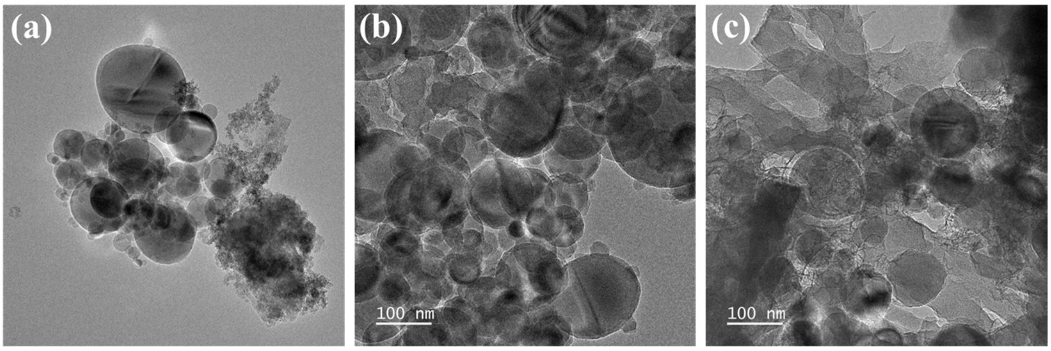


**Figure S2.** TEM images of (a) Si@C, (b) Si@PS, (c) Si@void@NCN-0.5.

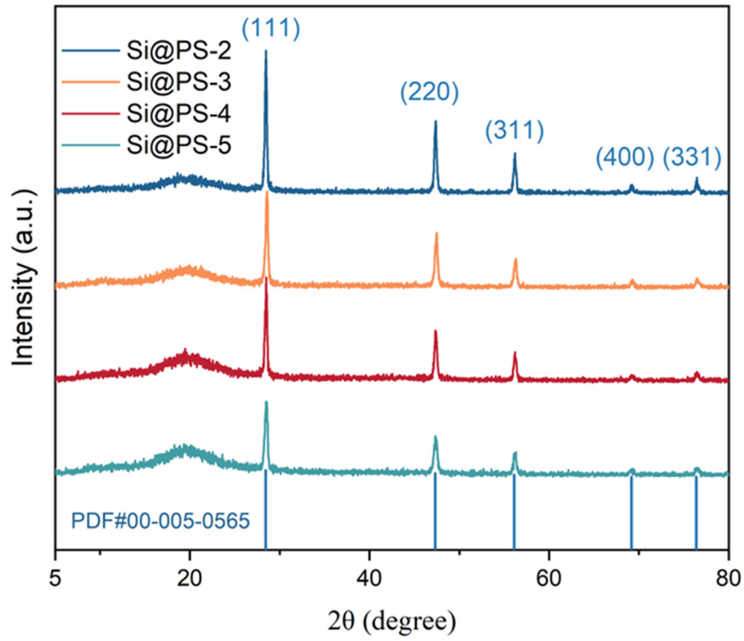


**Figure S3.** XRD patterns of samples with different PS ratios.

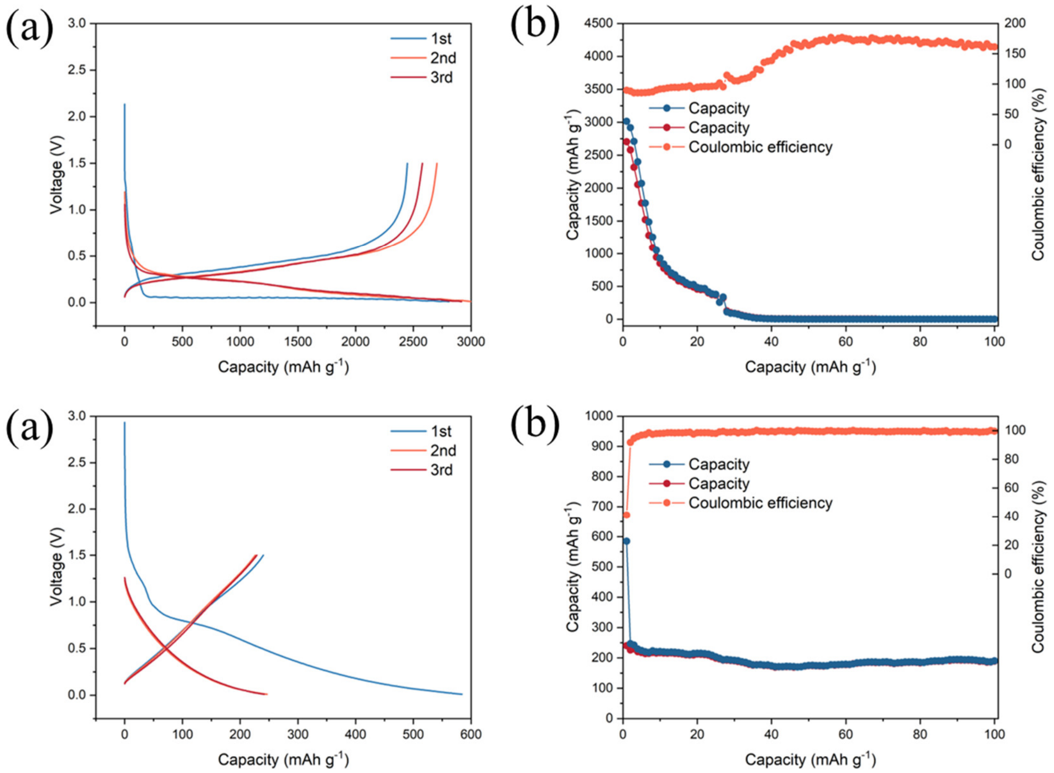


**Figure S4.** (a-b) Charge–discharge profiles and cycling performance of Si NPs and PANI at a current density of 100 mA g$^{-1}$: (a, b) Si NPs; (c, d) PANI.

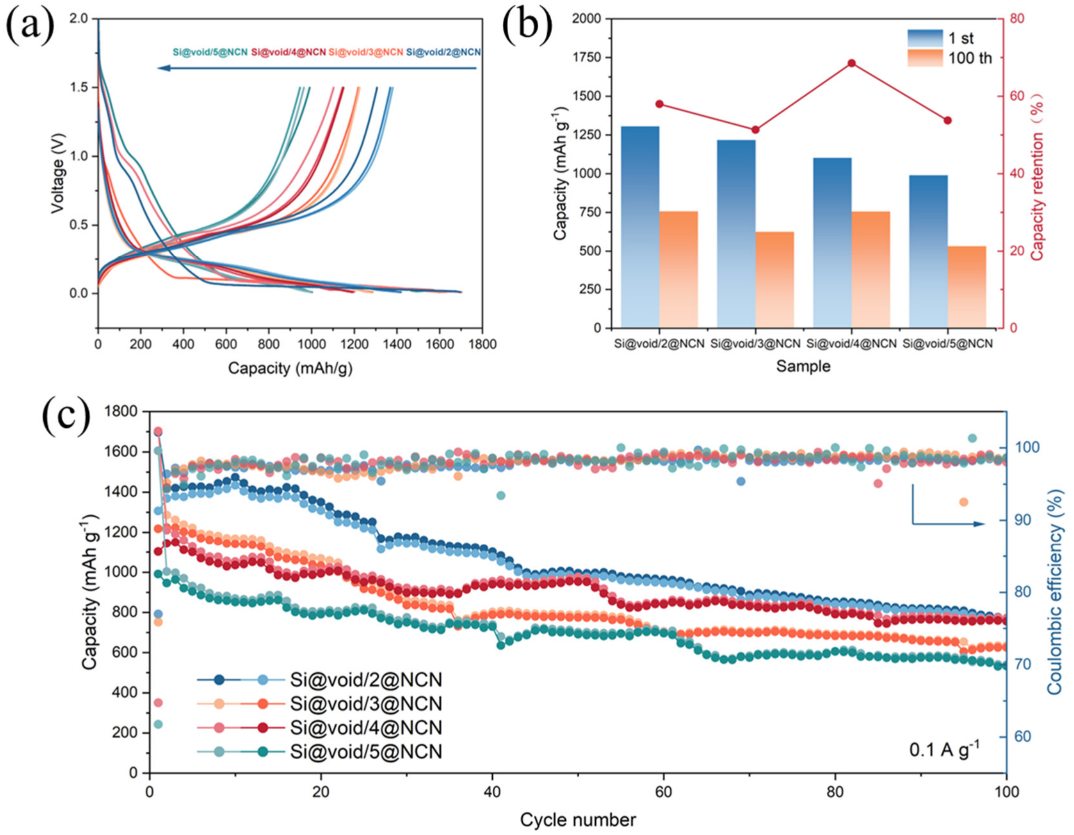


**Figure S5.** Samples with different PS coating ratios: (a) charge–discharge profiles, (b) capacity retention, and (c) cycling performance.

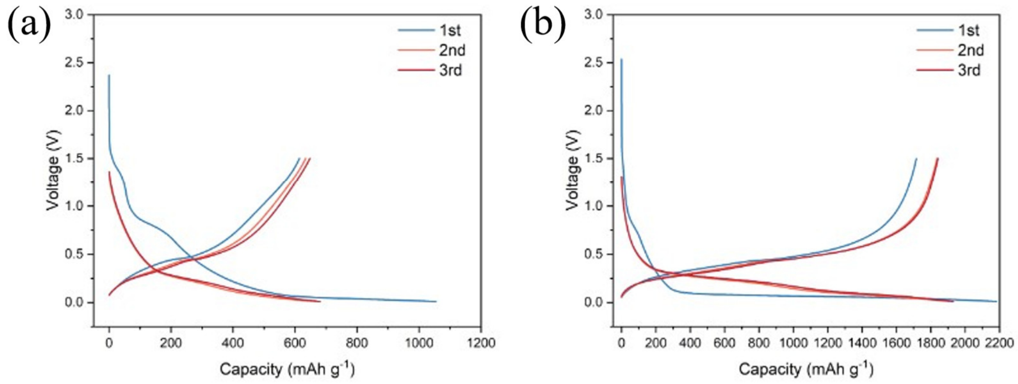


**Figure S6.** Charge–discharge profiles and cycling performance of (a) Si@void@NCN-1.5, (b) Si NPs.

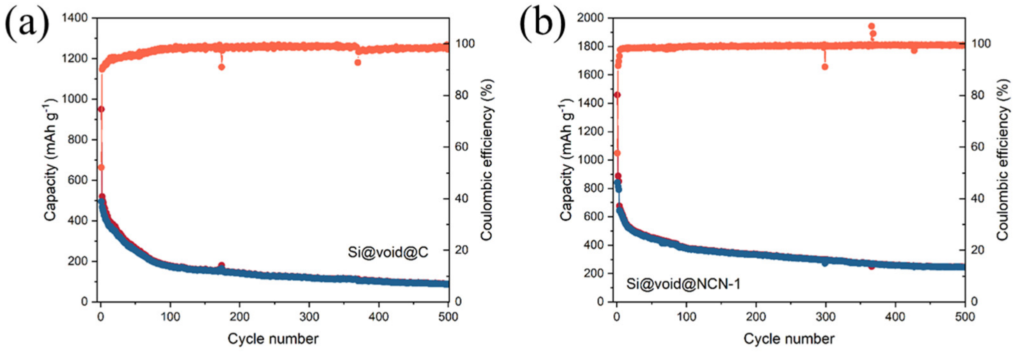


**Figure S7.** (a) Cycling performance comparison of the PAN-derived control sample with the same Si@PS ratio and carbon-source feeding amount, (b) Cycling performance of Si@void@NCN-1 carbonized at 700 °C.

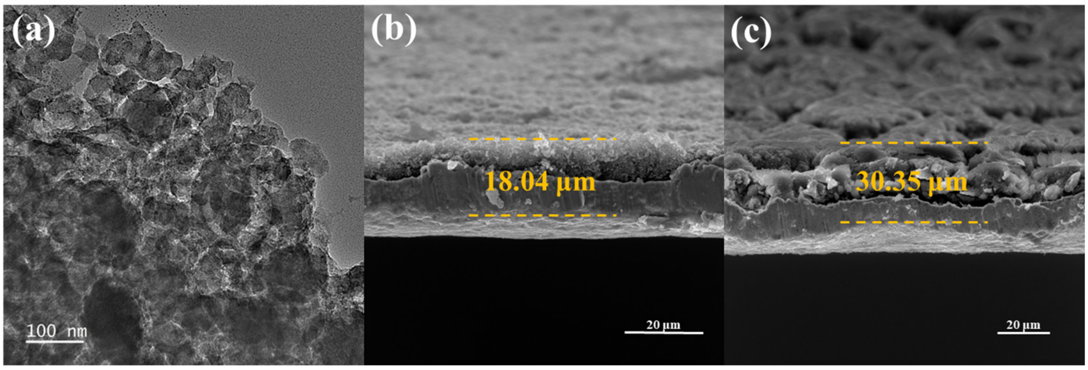


**Figure S8.** Post-rate-test morphology: (a) TEM image of the material, (b) cross-sectional image of the Si@void@NCN-1 electrode, and (c) cross-sectional image of the Si@C electrode.

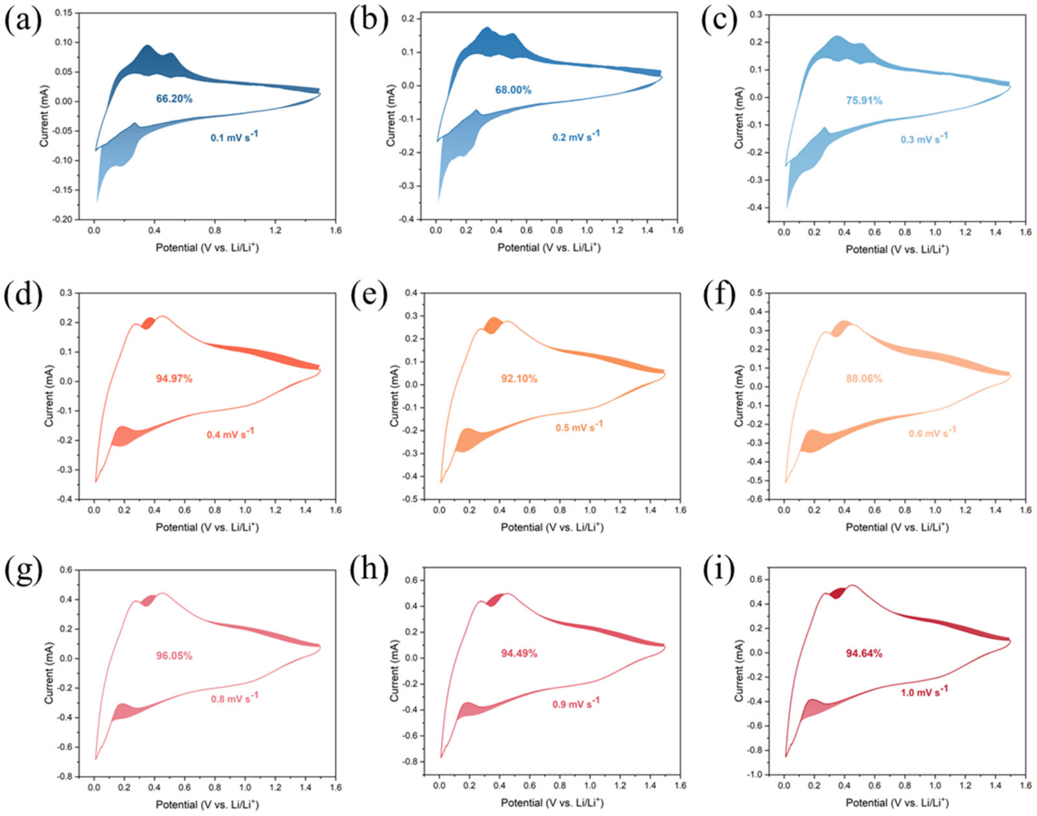


**Figure S9.** (a-i) The detailed pseudocapacitive contribution at different scan rates.

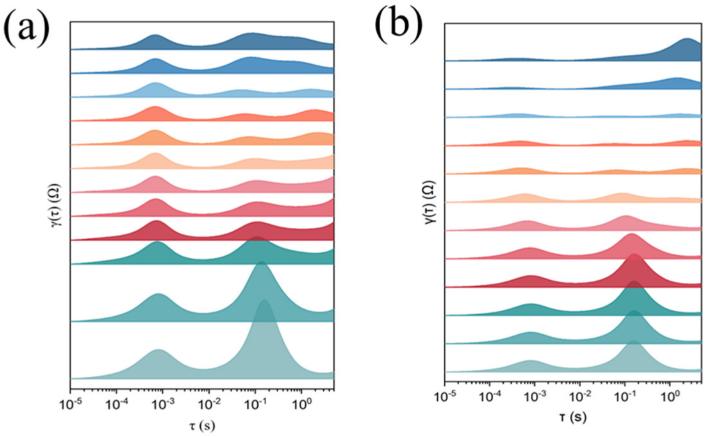


**Figure S10.** (a) The DRT curves during discharge of Si@void@NCN-1, and (b) the DRT curves during charge of Si@void@NCN-1.
